# Combined Effects of *in Utero* and Adolescent Tobacco Smoke Exposure on Lung Function in C57Bl/6J Mice

**DOI:** 10.1289/EHP54

**Published:** 2016-11-04

**Authors:** David Drummond, Mélissa Baravalle-Einaudi, Guillaume Lezmi, Shamila Vibhushan, Marie-Laure Franco-Montoya, Alice Hadchouel, Jorge Boczkowski, Christophe Delacourt

**Affiliations:** 1 INSERM (Institut National de la Santé et de la Recherche Médicale), U955, Equipe 04, IMRB (Institut Mondor de Recherche Biomédicale), Créteil, France; 2 Pneumologie Pédiatrique, Necker, AP-HP (Assistance Publique-Hôpitaux de Paris), France; Centre de Référence des Maladies Respiratoires Rares, Paris, France; 3 Université Paris-Descartes, Paris, France

## Abstract

**Background:**

Fetal determinants of airway function, such as *in utero* exposure to maternal cigarette smoke (CS), may create a predisposition to adult airflow obstruction and chronic obstructive pulmonary disease (COPD) in adulthood. It has been suggested that active smoking in adolescence and preexisting airflow obstruction have synergistic deleterious effects.

**Objective:**

We used a mouse model to investigate whether there is a synergistic effect of exposure to CS *in utero* and during adolescence on lung function.

**Methods:**

Female C57Bl/6J mice were exposed to CS or to filtered room air during pregnancy. Exposure to CS began 2 weeks before mating and continued until delivery. After birth, the pups were not exposed to CS until day 21 (D21). Between D21 and D49, corresponding to “adolescence,” litters were randomized for an additional 4 weeks of exposure to CS. Lung morphometry, lung mechanics, and the expression of genes involved in senescence were evaluated in different subsets of mice on D21 and D49.

**Results:**

*In utero* exposure to CS induced significant lung function impairment by D21. CS exposure between D21 and D49 induced significant functional impairment only in mice exposed to CS prenatally. On D49, no difference was observed between subgroups in terms of lung p53, p16, p21, and Bax mRNA levels.

**Conclusions:**

Our findings suggest that prenatal and adolescent CS exposure have a synergistic effect on lung function in mice. The combined effect did not appear to be a consequence of early pulmonary senescence.

**Citation:**

Drummond D, Baravalle-Einaudi M, Lezmi G, Vibhushan S, Franco-Montoya ML, Hadchouel A, Boczkowski J, Delacourt C. 2017. Combined effects of *in utero* and adolescent tobacco smoke exposure on lung function in C57Bl/6J mice. Environ Health Perspect 125:392–399; http://dx.doi.org/10.1289/EHP54

## Introduction

Chronic obstructive pulmonary disease (COPD) is a major cause of death and disability worldwide that is expected to increase in prevalence over the next few years (Lopez et al. 2006). A better understanding of the conditions leading to its occurrence is required to facilitate its prevention. Smoking is the main risk factor for COPD, but only a minority of smokers develop the disease (Chen et al. 2010), and nonsmokers account for a substantive part of the burden of COPD (Eisner et al. 2010). Thus, both environmental exposure and individual susceptibility factors are involved in the pathogenesis and progression of COPD.

The quality of lung development is known to play a critical role in determining pulmonary function in adults (Boucherat et al. 2016; Rennard and Drummond 2015). Previous findings suggest that the maximum lung function attained after childhood lung growth is a key determinant of the rate of functional decline leading to a COPD diagnosis (Lange et al. 2015; Rennard and Drummond 2015). Prenatal airway growth may be a key predictor of adult lung function. For example, poor airway function measured shortly after birth in 123 infants predicted airflow obstruction in early adulthood (Stern et al. 2007). A systematic review and meta-analysis by Kotecha et al. (2013) indicated that premature birth, which interrupts physiological prenatal growth, is negatively associated with later pulmonary function in children and adults. Finally, genes involved in early airway morphogenesis have been associated with lung function and COPD (Klar et al. 2011; Van Durme et al. 2010). The impairment of prenatal airway growth may be related to exposure to environmental factors, such as *in utero* exposure to maternal cigarette smoke (CS). Exposure to maternal CS has been negatively associated with lung function in adolescents and young adults (Hollams et al. 2014) and in early adulthood (Hayatbakhsh et al. 2009). One key question concerns the potential role of preexisting airway obstruction, secondary to impaired prenatal growth, as a risk factor for an earlier onset or more rapid decline in lung function in individuals beginning to smoke actively during adolescence or early adulthood. Previous studies have suggested that maternal smoking and personal smoking may have a synergistic effect on lung function decline, but these studies were unable to separate the influences of prenatal and postnatal exposure to parental smoking (Guerra et al. 2013; Upton et al. 2004).

In this study, we used a murine model to investigate whether *in utero* exposure to CS caused an accelerated decline in lung function in mice that were also exposed to CS during adolescence.

Our secondary objective was to determine whether the combined effects of *in utero* exposure and exposure during adolescence to CS led to an acceleration of lung senescence. Several studies have suggested that the aging process is accelerated in the development of COPD (Ito and Barnes 2009). Prebronchodilator forced expiratory volume in the first second (FEV1) values were positively associated with telomere length in peripheral leukocytes, a biomarker of cell senescence, in a combined analysis of 14 European cohorts (Albrecht et al. 2014).

## Methods

### Animals

All of the animal experiments were approved by the local institutional animal care and use committee (Agreement number 11/11/15-4), and all animals were treated humanely and with regard for alleviation of suffering. Twelve-week-old female C57Bl/6J mice were obtained from Janvier (France), housed in groups of 4, and given 1 week to acclimate to the housing facility. Environmental conditions were a temperature of 21°C, humidity of 55%, lighting of 300 lux (at bench level) and a 12:12 light:dark cycle with lights on at 0800 hours and off at 2000 hours. Animals were housed in 330 mm × 150 mm × 130 mm cages and given access to mouse maintenance food (reference A03.10, SAFE) and water *ad libitum*. During housing, animals were monitored twice daily for health status. After birth, pups were left in the cage of their mother until day 21. On day 21, litters were separated from their mothers. Males and females were separated, and same-sex mice were randomly housed in groups of 8, depending on the group (Smoke or Air) of their dam.

**Figure 1:**
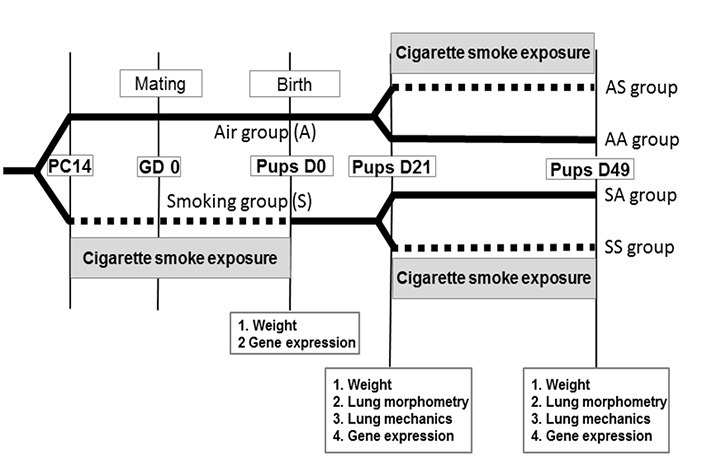
Study plan. The dotted lines indicate periods of exposure to cigarette smoke. At birth, 31 pups were analyzed in the Smoke group (weight *n* = 31, gene expression *n* = 24) and 29 in the Air group (weight *n* = 29, gene expression *n* = 21). On day 21, pooling two experiments (see Figures S1 and S2), 25 pups were analyzed in the Smoke group (weight *n* = 25, lung morphometry *n* = 17, lung mechanics *n* = 14, gene expression *n* = 8) and 26 in the Air group (weight *n* = 26, lung morphometry *n* = 10, lung mechanics *n* = 15, gene expression *n* = 8). On day 49, 11 pups were analyzed in the SS group (weight *n* = 11, lung morphometry *n* = 8, lung mechanics *n* = 8, gene expression *n* = 11), 11 in the SA group (weight *n* = 11, lung morphometry *n* = 8, lung mechanics *n* = 8, gene expression *n* = 11), 19 in the AS group (weight *n* = 19, lung morphometry *n* = 17, lung mechanics *n* = 8, gene expression *n* = 16), and 12 in the AA group (weight *n* = 12, lung morphometry *n* = 10, lung mechanics *n* = 8, gene expression *n* = 7). AA, Air–Air; AS, Air–Smoke; GD0, gestational day 0 (conception); PC14, pre-conception day 14 (14 days before conception); Pups D0, pups aged 0 day (birth); Pups D21, pups aged 21 days; Pups D49, pups aged 49 days; SA, Smoke–Air; SS, Smoke–Smoke.

Animals in the CS exposure group were exposed to filtered mainstream smoke (corresponding to the smoke from 3R4F research cigarettes with filters from the Kentucky Tobacco Research and Development Center, University of Kentucky) 9 cigarettes/hr, 2 hr/day, with a smoking machine (Anitech, Paris, France). Animals were placed in a restraining box, and CS was delivered cyclically (1 puff/min, 15 sec with the box closed and the remaining 45 sec with the box open, to mimic typical smoking behavior as closely as possible). A similar procedure was used for control animals except that they were exposed to filtered room air rather than to CS. Dams were exposed to CS or filtered air for 2 hr/day, 7 days/week, beginning 2 weeks before mating and continuing through the 3 weeks of gestation until delivery. For Experiment 2 (see below), a subset of pups were randomized (across litters) to receive CS exposure from D21 through D49, a period corresponding to adolescence [puberty occurs between days 35 and 50 in C57Bl/6J mice (Bates et al. 2003)] (Figure 1). No pups were exposed to CS from birth (D0) to D21, which corresponds to the period of lung growth.

Exposure to CS was monitored through recurrent measurements of blood carboxyhemoglobin (HbCO) levels in dams before mating (preconception day 10), during pregnancy (gestational day 5), and in pups 2 days after the beginning (day 23) and 1 day before the end (day 48) of the exposure period. Blood (0.2 mL) was collected within 5 min following the 2-hr CS exposure from an animal randomly selected from each of the five restraining boxes, and blood HbCO levels were assessed using a Radiometer ABL 700 blood gas analyzer (Brønshøj, Denmark). The median HbCO levels measured at different time points ranged from 20.9% to 32.5% for dams exposed to CS and from 24.1% to 31.2% for pups exposed to CS, compared with 0.7% to 1.3% and 0.7% to 1.1% for dams and pups exposed to filtered air, respectively (see Table S1).

Two experiments were conducted for this study. In Experiment 1 (see Figure S1), we compared pups with and without *in utero* exposure to CS, including weight and gene expression at D0 (four litters in each group, 31 and 29 pups, respectively), and weight, gene expression, and lung morphometry at D21 (two litters and 11 pups in the CS group; three litters and 11 pups in the filtered-air group). In Experiment 2 (see Figure S2), we measured weight, lung morphometry, and lung mechanics on D21 in mice with and without *in utero* exposure to CS (14 and 15 pups, respectively), and we measured weight, gene expression, lung morphometry, and lung mechanics on D49 in four subgroups of mice:

Air–Air (AA), mice never exposed to CS (*n* = 12)
Air–Smoke (AS), exposed to CS only between D21 and D49 (*n* = 19)
Smoke–Air (SA), exposed to CS only during fetal development (*n* = 11)
Smoke–Smoke (SS), exposed to CS throughout fetal development and between D21 and D49 (*n* = 11).

For each experiment and in each group, offspring from at least two different litters were assessed. The experimenters were blinded to the exposure groups while processing data.

### Lung Mechanics

All functional measures were performed using the flexiVent™ system (SCIREQ, Montreal, PQ, Canada)—an invasive method that directly measures pulmonary functions via the use of a preprogrammed ventilator and system-specific maneuvers. This technology measures standard and maximal pressure–volume (PV) curves, resulting in clinically relevant parameters, such as vital capacity, resistance, and compliance of the respiratory system. Data obtained using this method were recently shown to distinguish BALB/c mice models of emphysema and of lung fibrosis from controls (Vanoirbeek et al. 2010). We assessed lung mechanics with the flexiVent™ system on D21 and D49. Mice were anesthetized with an intraperitoneal injection of ketamine and xylazine (130 mg/kg ketamine and 8.5 mg/kg xylazine), followed by an additional injection of pentobarbital (40 mg/kg) to prevent spontaneous breathing. A tracheotomy was performed, and the animals were connected to the flexiVent™ system and quasi-sinusoidally ventilated with a tidal volume of 10 mL/kg at a frequency of 150 breaths/min and a positive end-expiratory pressure of 2 cm H_2_O. The flexiVent™ ventilator introduced force oscillation measurements through a graphite piston system that controlled circuit pressure/volume displacement. First, a maximal vital capacity perturbation corresponding to total lung capacity (TLC) was used to obtain maximal inflation of the lungs to a standard pressure of +30 cm H_2_O, and the lungs were then deflated. A ‘‘snapshot perturbation’’ maneuver was then used to measure the resistance (R), compliance (C), and elastance (E) of the whole respiratory system. A forced oscillation perturbation (‘‘Quick Prime’’) was used to obtain airway resistance (Rn), tissue damping (resistance) (G), tissue elasticity (H), and tissue hysteresivity (G/H). Finally, maximal PV loops between +30 cm H_2_O and –30 cm H_2_O were generated to obtain static compliance (Cst) and static elastance (Est). For each parameter, an average of three measurements was calculated and depicted per mouse. A coefficient of determination of 0.95 was the lower limit for accepting a measurement.

### Lung Morphometry

The left lung was fixed with 4% paraformaldehyde at a constant hydrostatic pressure of 20 cm H_2_O for at least 30 min. Lung volumes were estimated by water displacement, as previously described (Scherle 1970). The lungs were dehydrated and embedded in paraffin, and frontal sections were stained with hematoxylin and eosin (H&E). We evaluated a minimum of eight random fields from each left lung of every offspring at 10× magnification by microscopic projection with the Zen program (Zeiss, Germany). We used the indirect stereological method to quantify mean linear intercept (L_m_), lung alveolar surface area, and surface–to–volume ratio, as previously described (Knudsen et al. 2010). The mean linear intercept (L_m_) is commonly used as an index for characterizing the enlargement of airspaces in emphysema (Parameswaran et al. 2006).

To study airway remodeling, we used the Sirius Red staining technique (Malkusch et al. 1995) for quantitative morphometric assessment of collagen content. With this technique, collagen fibers are stained bright red, and nuclei/cytoplasm are bright yellow. Collagen deposition in isolated bronchi was assessed by color segmentation (with the “colour deconvolution” plug-in, see “Algorithms used with Image J software” in the Supplemental Material) which transformed the RGB (red, green, blue) image into grayscale. Collagen deposition areas that were previously stained red turned black, allowing quantification of the surface area of collagen deposition per unit of the airway basement perimeter.

### Gene Expression

Previous reports have shown that cigarette smoke induces airway epithelial cellular senescence (Tsuji et al. 2004) and that accelerated alveolar cell senescence was found in patients with COPD (Tsuji et al. 2006). The expression of genes associated with senescence (p16, p21, and p53) and with apoptosis (p53 and Bax) were studied. p16 and p21 are two senescence-associated cyclin-dependent kinase inhibitors. Their expression is increased in most senescent cells (Krishnamurthy et al. 2004), including alveolar type II and endothelial cells of patients with COPD (Tsuji et al. 2006). p53 is a tumor suppressor that is involved in senescence through the induction of p21, and in apoptosis through the activation of the proapoptotic Bcl-2 protein Bax (Chipuk et al. 2004). Mutant mice with activated p53 display an early onset of phenotypes associated with aging (Tyner et al. 2002).

Levels of p16, p21, p53, and Bax mRNA were analyzed at birth and then on days 21 and 49. Total lung RNA was extracted with the RNeasy Protect Mini Kit (Qiagen, France) and reverse transcribed. Quantitative polymerase chain reaction (qPCR) was performed using an ABI Prism 7000 Sequence Detection system (Thermo Fisher Scientific, Waltham, MA) with Platinum SYBR Green (Invitrogen, Carlsbad, CA). For each sample, the expression of the gene of interest was normalized against the geometric mean value for the housekeeping genes splicing factor 3a subunit 1 (*Sf3a1*) and hypoxanthine phosphoribosyltransferase 1 (*hprt1*). RNA levels in mouse lung were quantified by a relative quantification method (the ΔΔCT method), as previously described (Livak and Schmittgen 2001). The primer sequences used are reported in Table S2. One biological sample was used per experimental condition, and each sample was analyzed in triplicate.

Finally, lung immunohistochemistry was performed to corroborate the increase in p16 gene expression in smoke-exposed lung that had been detected at day 21 by qPCR. Sections were prepared and assayed as described in “Lung immunohistochemistry” in the Supplemental Material. Ten digital photomicrographs at 10× magnification were obtained for a histologic section of each sample. Color segmentation and ImageJ software with the “colour deconvolution” plug-in (http://imagej.nih.gov/ij/; National Institutes of Health, Bethesda, Maryland, USA, 1997–2016) was used to quantify p16 expression relative to the surface area of the cell nucleus (see “Algorithms used with Image J software” in the Supplemental Material).

### Statistical Analysis

Data were analyzed on an individual dam/pup basis using GraphPad Prism v.5.03 (La Jolla, CA). Median values and interquartile ranges are reported. Comparisons were performed with Mann–Whitney tests or Kruskal–Wallis nonparametric analysis of variance tests followed by post hoc Dunn tests for four groups.

## Results

### Impact of Maternal CS Exposure during Pregnancy

There was no significant difference in fertility between females that were or were not exposed to CS [13 of 40 (32.5%) and 14 of 40 (35%), respectively, found to be pregnant after mating] (see Figures S1 and S2). The median number of offspring per litter also did not differ significantly between CS-exposed and air-exposed females [9; interquartile range (IQR): 6–10 and 7; IQR: 5–8.5, respectively]. In addition, there were no significant differences in maternal body weight at baseline (preconception day 14) or on GD0, GD12, or GD18 (see Table S3).

### Effect of Prenatal CS Exposure on Offspring

At birth, mice that were exposed to CS prenatally (*n* = 31) had significantly lighter body weights than controls (*n* = 29) [median (IQR) of 1.1 g (1.1–1.2) and 1.3 g (1.3–1.4), respectively (*p* < 0.001)] (see Table S4). On D21, body weights were not significantly different between pups with (*n* = 47) and without (*n* = 57) prenatal CS exposure [median (IQR) 10.0 g (8.6–11.3) and 10.7 g (9.4–11.7), respectively]. A subset of 27 mice (17 from the CS group and 10 from the control group) was analyzed for lung volume and lung morphometry on D21. Despite a similar median weight in these subsets of mice, lung volume was significantly smaller in CS mice than in control mice (Table 1, *p* = 0.02).

**Table 1:** Lung volume and lung morphometry of the subsets of mice analyzed on these parameters on day 21 and day 49, with their corresponding weights.

Morphometry	D21 *In utero* exposure			D49 *In utero* + postnatal exposure				
	A *n *= 10^*a*^	S *n *= 17^*a*^	*p*	AA *n *= 10^*b*^	AS *n *= 17^*b*^	SA *n *= 8^*b*^	SS *n *= 8^*b*^	*p*
Weight (g)	11.0 (9.6–11.8)	10.7 (9.4–12.5)	0.88	18.2 (16.8–21.9)	18.5 (16.7–20.0)	19.0 (16.6–21.3)	16.2 (15.4–17.1)	0.05^*d*^
Lung volume (mL)	0.44 (0.41–0.47)	0.39 (0.34–0.44)	0.02	1.8 (1.6–2.2)	1.7 (1.6–1.9)	1.7 (1.6–2.0)	1.7 (1.5–1.8)	0.65^*d*^
Mean linear intercept (μm)	17.3 (16.6–19.0)	18.6 (16.2–20.0)	0.74	15.4 (15.2–15.7)	16.10 (15.5–16.4)	16.17 (14.7–17.0)	16.40 (15.6–17.7)	0.16^*d*^
Lung alveolar surface area (cm^2^)	84 (71–95)	84 (68–92)	0.94	434 (363–532)	411 (359–443)	422 (352–533)	357 (334–413)	0.20^*d*^
Surface to-volume ratio (cm^–^^1^)	285 (263–307)	272 (262–292)	0.41	277 (268–282)	263 (259–283)	271 (248–296)	261 (253–273)	0.57^*d*^
Collagen deposition^*c*^ [value/bronchus perimeter (μm^–1^)]	—	—	—	2.83 (1.84–4.12)	—	—	3.5 (2.96–3.66)	0.46
Notes: A, air; D21, day 21; D49, day 49; S, smoke. Median values and interquartile range are reported. ^***a***^On D21, 2 and 4 mice in the in the control and S group from experiment 1, respectively, and 8 and 13 mice in the control and S group from experiment 2, respectively, were analyzed for lung volume and morphometry. The results presented in this table for D21 correspond to the pool of these 10 and 17 mice. ^***b***^On D49, all the mice belonged to experiment 2. Ten mice from the AA group [never exposed to cigarette smoke (CS)], 17 mice from the AS group (exposed to CS only between D21 and D49), 8 mice from the SA group (exposed to CS only during fetal development), and 8 mice from the SS group (exposed to CS throughout fetal development and between D21 and D49) were analyzed for lung volume and lung morphometry. The corresponding weights of these subsets of mice are presented in the table. ^***c***^Collagen deposition was measured in 8 mice from the SS group and 8 mice from the AA group on D49. ^***d***^Pairwise statistical comparisons were performed, and no statistically significant difference was found.								

Alveolarization was evaluated measuring mean linear intercept, alveolar surface area, and the surface-to-volume ratio. No significant differences were observed for these parameters between CS and control mice at D21 (Table 1), which suggests that the smaller lung volume at D21 in mice exposed prenatally to CS was not attributable to hypoalveolarization.

**Figure 2:**
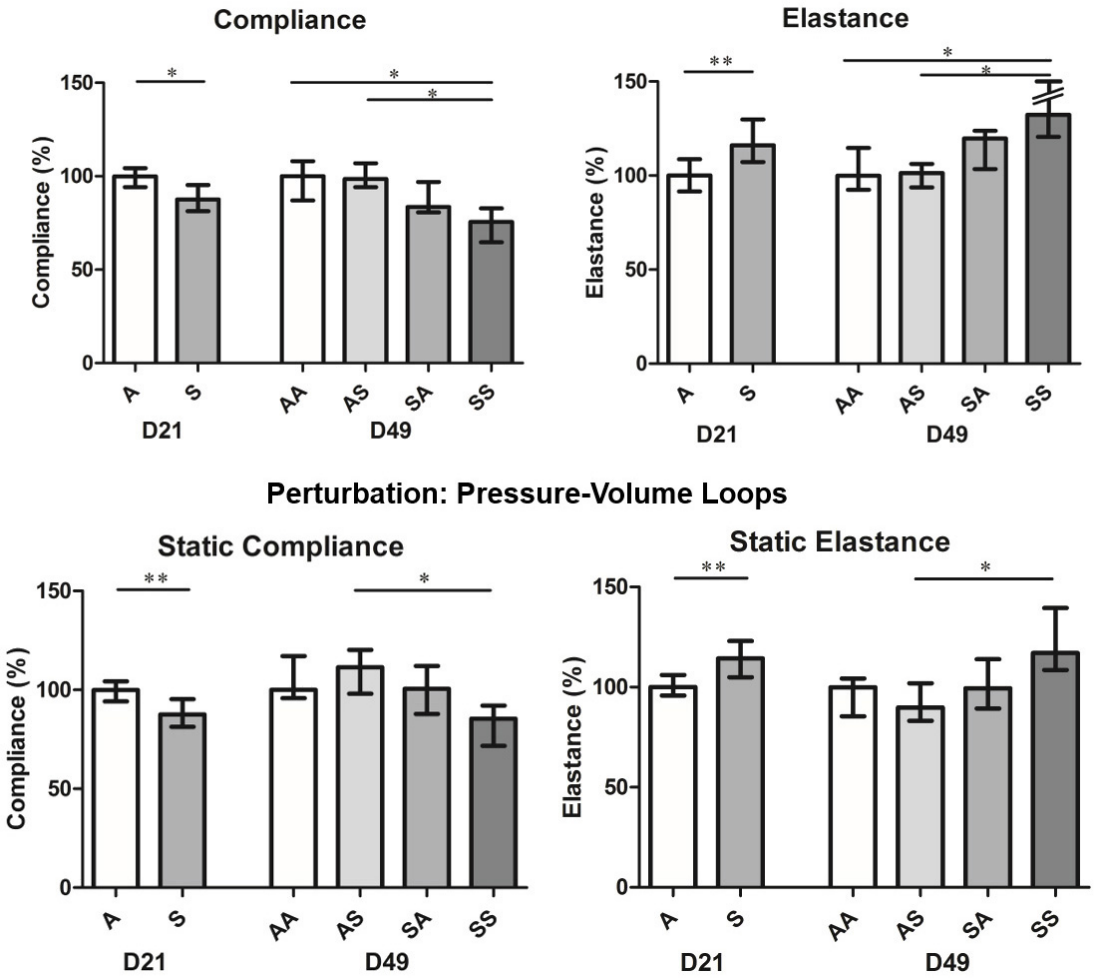
Lung mechanics on days 21 and 49. Results are presented as the median and interquartile range (IQR). Air group, *n* = 15; Smoke group, *n* = 14; Air–Air group, Air–Smoke group, Smoke–Air group, Smoke–Smoke group, *n* = 8 per group. Numeric data for this figure are available in Tables S5 and S6. On D21, medians were compared using a Mann–Whitney test. Differences were statistically significant between Smoke and Air groups regarding compliance (*p* = 0.01), elastance (*p* < 0.01), static compliance (*p* < 0.01) and static elastance (*p* < 0.01). On D49, medians were compared using a Kruskal–Wallis test followed by post-hoc Dunn tests for four groups. Overall *p*-values (not shown on the figure) were < 0.01 for compliance and elastance and < 0.05 for static compliance and static elastance. Post-hoc Dunn tests revealed statistically significant differences between the SS group and the AA and AS groups for compliance and elastance (*p* < 0.05) and between the SS and AS groups for static compliance and static elastance (*p* < 0.05). *p < 0.05; **p < 0.01. A, air; AA, Air–Air; AS, Air–Smoke; D21, day 21; D49, day 49; SA, Smoke–Air; SS, Smoke–Smoke.

Lung function was found to be significantly impaired on D21 in mice exposed prenatally to CS (Figure 2; see also Tables S5 and S6). These mice had a significantly lower compliance (*p* = 0.01) and a significantly higher elastance (*p* < 0.01) of the whole respiratory system than control mice. These results were confirmed by pressure–volume loops, which showed static compliance to be significantly lower (*p* < 0.01) and static elastance to be significantly higher (*p* < 0.01). Furthermore, the constant-phase model (Quickprime) showed tissue elasticity to be significantly higher (*p* = 0.02) (see Table S5). The results obtained were not significantly different between male and female mice exposed to air, or between male and female mice exposed to smoke (see Table S7). Resistance was higher in mice with prenatal exposure to CS (median 1.51 cm H_2_O.sec/mL, IQR 1.35–1.65) than in control mice (median 1.31 cm H_2_O.sec/mL IQR 1.16–1.76), but the difference was not significant (*p* = 0.47) (see Table S5).

### Combined Effect of Prenatal and Postnatal CS Exposure in Mice

On D49, significantly lower body weights were observed in the SS group than in the AA and AS groups (see Table S4).

Several lung function values differed significantly between the SS group and the AA and AS groups, whereas there were no significant differences between the AA and AS groups (Figure 2; see also Tables S5 and S6). This finding suggests that effects of CS exposure during adolescence on lung function were limited to mice that also had prenatal CS exposure, consistent with a synergistic effect. Compliance and static compliance values were lowest in the SS group, and elastance and static elastance values were highest in the SS group.

**Figure 3:**
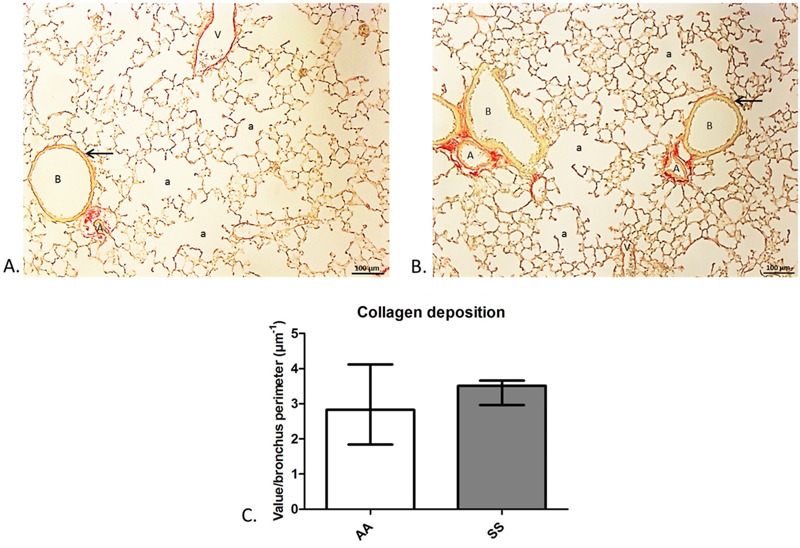
Collagen deposition in the main bronchi. Histologic analysis of lung parenchyma of 49-day-old mice (*A*) from the Air–Air group, and (*B*) from the Smoke–Smoke group. Collagen deposition in the airway wall was visible with picrosirius red staining (arrow). (*C*) Collagen deposition in the main bronchi did not differ significantly between the Air–Air and Smoke–Smoke groups (*n *= 8 per group). a, alveolus; A, artery; B, bronchus; V, vein.

In the microscopic images, morphometric parameters were not significantly different across the four exposure groups (Table 1). The mean linear intercept was higher and lung alveolar surface area was lower in the SS group than in the other groups, but the difference was not statistically significant (Table 1). Similarly, median collagen deposition was higher in the SS group than in the AA group, but the difference was not statistically significant (Table 1 and Figure 3). These findings suggest that associations between CS exposure and reduced lung function were not mediated by emphysema or fibrosis.

### Lung Expression of Senescence Genes

**Figure 4:**
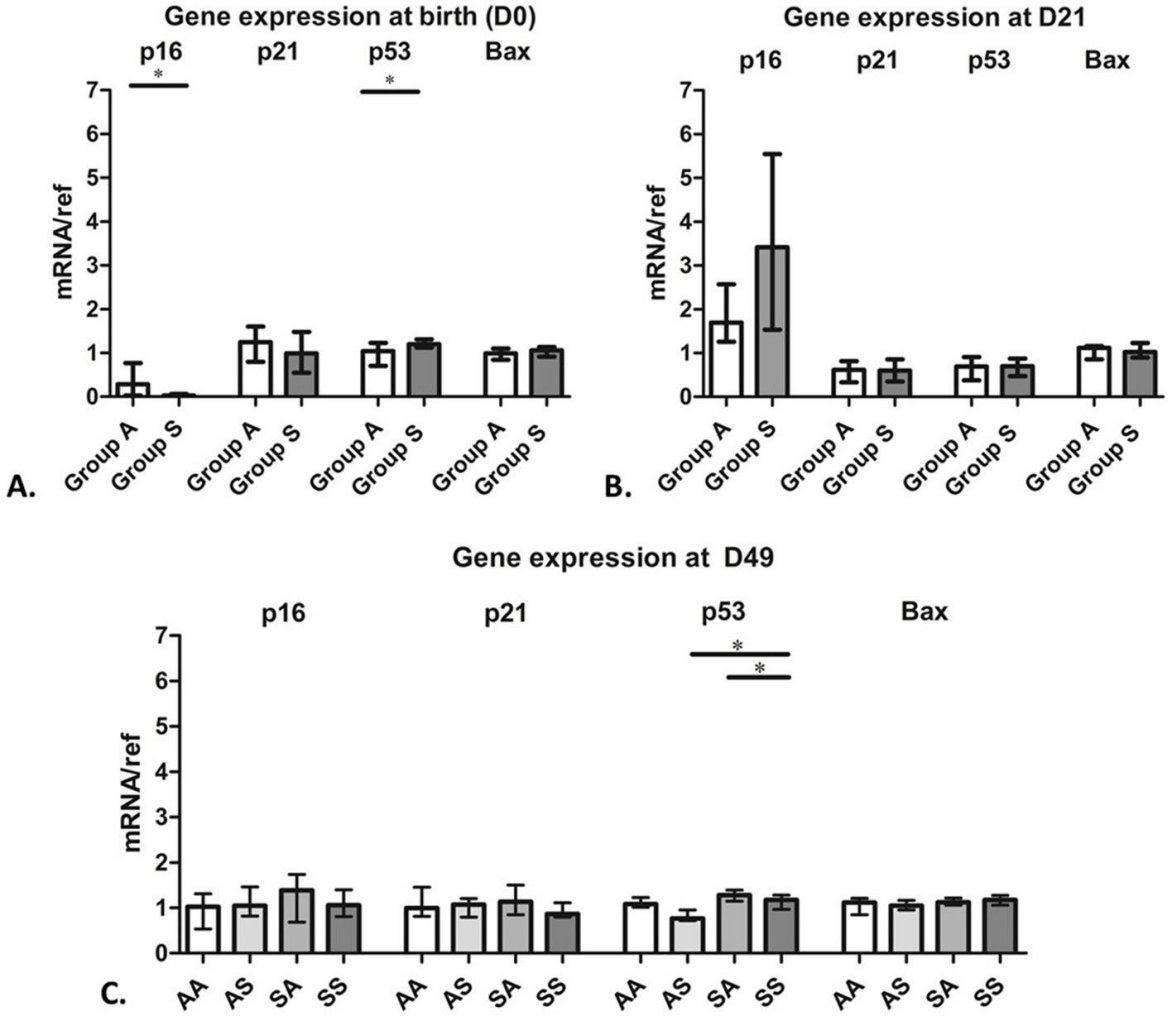
Expression of the genes for p16, p21, p53, and Bax at birth and on days 21 and 49. Results are presented as the median and interquartile range (IQR). (*A*) Gene expression at birth after prenatal exposure to air (Group A) or cigarette smoke (CS) (Group S). Group A, *n* = 21; Group S, *n *= 24; *p < 0.05. (*B*) Gene expression on day 21 (D21) after prenatal exposure to air (Group A) or CS (Group S); *n *= 8 per group. (*C*) Gene expression on day 49 (D49) in mice that were never exposed to CS, (AA, *n *= 7), exposed to CS during postnatal life only (AS, *n *= 16), exposed during prenatal development only (SA, *n *= 11), and exposed during prenatal development and postnatal life (SS, *n *= 11), *p < 0.05.

Neither prenatal nor postnatal CS exposure was consistently associated with the expression of genes involved in senescence pathways (p16, p21, p53, and Bax). Lung p53 mRNA levels were significantly higher at birth in mice exposed prenatally to CS than in controls (Figure 4A). p53 expression was also higher in SA (*n* = 11) and SS (*n* = 11) mice than in AA (*n* = 7) or AS (*n* = 16) mice on D49, although the differences were not significant for AA mice (Figure 4C). Lung p16 mRNA levels were low at birth in most mice, similar to the expression of other genes on D49, and high relative to those of the other genes on D21 (Figure 4A–C). The median p16 value on D21 was higher in pups exposed to maternal tobacco than in control pups, but the difference was not significant. However, lung p16 protein levels on D21 were significantly higher in mice with prenatal CS exposure than in controls (*p* = 0.02) (see Figure S3C).

Overall, these findings suggest that effects of prenatal and adolescent CS exposure on lung function are not a consequence of early pulmonary senescence.

## Discussion

Maternal smoking during pregnancy is known to be associated with poor lung function at birth (Stick et al. 1996) and during adolescence and early adulthood (Hollams et al. 2014; Svanes et al. 2004). Some studies in humans have suggested that parental and personal smoking may have a synergistic effect on the risk of COPD (Guerra et al. 2013; Upton et al. 2004), but a causal link has not been confirmed. To our knowledge, our study is the first to demonstrate evidence of a synergistic effect of prenatal and postnatal CS exposure on lung function in early adulthood in a murine model.

Our mouse model of fetal and adolescent exposure to smoking produced acute HbCO levels in dams and pups that were comparable to those measured after water-pipe smoking in humans (Bentur et al. 2014), and the number of cigarettes pups were exposed to between D21 and D49 was comparable to the consumption of cigarettes by regular young smokers [Australian Institute of Health and Welfare (AIHW) 2013; Fuller 2013]. However, our HbCO levels were higher than those observed in regular smokers (Bureau et al. 1982; Pojer et al. 1984; Russell et al. 1976) because in our model, as in most mouse models of tobacco exposure (Fallica et al. 2014; Ng et al. 2006, 2009; Penn et al. 2007; Singh et al. 2013; Wu et al. 2009), mice were exposed to CS continuously for 2 hr rather than intermittently as are human smokers.

Furthermore, our mouse model of fetal exposure to maternal smoking reproduced several consequences of *in utero* smoke exposure in humans, with lower birth weight and impaired lung function shortly after birth. An analysis of data from almost 17,000 singleton births in England, Scotland, and Wales in 1958 indicated that neonates whose mothers smoked after the fourth month of pregnancy had lower birth weights than other children (Butler et al. 1972), and a study of 663 children reported that lung function parameters measured 2–3 days after birth were lower in children whose mothers smoked during pregnancy, with significant associations in girls (Lødrup Carlsen et al. 1997). Maternal smoking during pregnancy was also associated with reduced lung function at 14 years of age in a study of 1,127 children (Hollams et al. 2014). Fetal exposure to nicotine has been shown to have many effects on lung structures and lung metabolism (Maritz and Harding 2011). In particular, prenatal tobacco exposure is known to alter airway structure: in a study of 32 infants that died of sudden infant death syndrome (SIDS), the distance between alveolar attachments in the intraparenchymal airways was larger in children whose mothers smoked during pregnancy than in the 8 children whose mothers did not smoke, which suggests that airways may have been narrower in the children exposed to maternal smoking (Elliot et al. 2003). In mice, prenatal nicotine exposure has been shown to modify airway geometry in offspring, increasing airway length and decreasing airway diameter, through alpha7 nicotinic acetylcholine receptor (nAChR)–mediated signals (Wongtrakool et al. 2012). We found no significant difference in alveolarization on day 21 (i.e., at the end of bulk alveolarization) between mice with and without prenatal exposure to tobacco smoke, but measures of pulmonary function were significantly lower in exposed mice, consistent with impaired airway growth. We observed significantly lower compliance and higher elastance on D21 in mice with prenatal exposure compared with controls, but there was no significant difference in airway resistance. Tomioka et al. (2002) reported a similar pattern in a mouse model of asthma, in which bronchoconstrictive responses to metacholine and inflammation occurred predominantly at the periphery of the lung, with a significant increase in tissue elastance but not airway resistance.

After a 4-week period of CS exposure during a period corresponding to adolescence in mice, lung function parameters were significantly lower in mice exposed prenatally to CS, but not in mice with adolescent exposure only, when compared with mice that were not exposed during either time period. These findings are consistent with the existence of a synergistic effect between prenatal and postnatal exposure to CS. Because we did not monitor CS exposure chamber conditions such as carbon monoxide or total suspended particulate matter, we could not determine whether there was a threshold effect regarding the amount of exposure needed to induce these alterations in lung function. Synergistic effects of parental and active smoking on lung function impairment have been suggested by human observational studies, but it has not been possible to separate potential effects of prenatal and postnatal environmental factors. Upton et al. (2004) reported that a 10 cigarette/day increase in maternal smoking during pregnancy was associated with significantly lower forced expiratory volume in one second (FEV1)/forced vital capacity (FVC) ratio in current smokers but not in never smokers or former smokers (Upton et al. 2004). Using the data from the Tucson cohort, Guerra and coworkers found that at the age of 26, participants with exposure to parental and active smoking had lower FEV1/FVC levels and a steeper decline in FEV1/ FVC between the ages of 11 and 26 than those not exposed to parental or active smoking (Guerra et al. 2013).

The impaired lung function observed in young adult mice was not associated with morphological alterations. In particular, we observed no airspace enlargement compatible with emphysema in the SS group, as indicated by mean linear intercept measurements. This may be due to the short duration of postnatal cigarette smoke exposure, 4 weeks, whereas at least 6 months of exposure are usually required to induce emphysema lesions in mice (Rinaldi et al. 2012). In adult mice exposed to CS, lung function impairment also precedes morphological alterations (Rinaldi et al. 2012). In another mouse study, an increase in airway resistance was reported after 2 months of exposure (Biselli et al. 2011), but we are not aware of any previous studies that measured resistance after only 1 month of exposure. In the present study, we evaluated the combined effects of prenatal CS exposure with adolescent exposure over a 4-week period that was not sufficient to induce lung function impairment in the absence of prenatal exposure. Our findings provide support for the hypothesis that prenatal developmental factors play a role in the early occurrence of COPD in humans. In a clinical trial that enrolled young adult smokers with mild to moderate airflow obstruction, participants with a greater degree of airflow obstruction at baseline had a more rapid rate of lung function decline over time (Drummond et al. 2012). Our findings also suggest that an accelerated decline in lung function may start shortly after the onset of smoking in adolescents at risk (i.e., those with impaired prenatal airway development).

We evaluated various mechanisms that could potentially account for our results. Prenatal and adolescent tobacco exposure did not appear to have a synergistic effect on lung structure, and morphometric measurements did not differ significantly among the subgroups. We evaluated collagen deposition in the airways because *in utero* exposure to CS has been associated with collagen deposition in the airways of monkey and mouse fetuses (Blacquière et al. 2009). No difference was observed between the AA and SS groups. However, it was not possible to study collagen deposition in the small airways. Small airway disease is a key factor leading to airway obstruction in early COPD and might account for the changes in lung function without accompanying morphological changes to the parenchyma observed in the SS group.

We hypothesized that impaired lung function in the SS group might be related to molecular markers of early pulmonary senescence. COPD is often considered to be a disease caused by accelerated lung aging (Ito and Barnes 2009), and lung aging is associated with a decrease in small-airway diameter (Niewoehner and Kleinerman 1974) and decreases in FEV1 and FVC (Janssens et al. 1999). Because *in utero* exposure to CS causes oxidative stress, a well-known inducer of cell senescence (van Deursen 2014), we hypothesized that lung aging might begin during prenatal development. However, our results did not support this hypothesis because p16, p21, and p53 mRNA levels, which were evaluated as molecular markers of senescence in lung tissue, were similar in all groups. Zhou et al. (2013) reported that the effects of 6 months of daily cigarette smoke exposure on increased p21 expression and emphysema in female C57Bl6 mice did not differ between mice whose exposure began at 3 months of age and those whose exposure began at 12 months of age, and the authors concluded that the age of the animal had no effect on emphysema development or on small-airway remodeling in response to cigarette smoke exposure (Zhou et al. 2013).

Finally, we did not study potential epigenetic changes, which might have accounted for some of our results. *In utero* exposure to CS has been associated with DNA methylation in humans [reviewed by Suter et al. (2013)]. Cigarette smoking was associated with differences in the methylation patterns of individual genes, including p16, in a study population of non–small cell lung cancer patients and heavy smokers (Destro et al. 2004), and methylation of 7 CpG sites within the p16 gene was significantly correlated in 120 paired maternal–offspring blood samples (Kile et al. 2010). Therefore, lower lung p16 mRNA levels at birth in pups with prenatal cigarette smoke exposure might reflect a transient effect on hypermethylation of the p16 promoter that was not apparent in mice evaluated on D21.

## Conclusions

Our results suggest that prenatal CS exposure and CS exposure during adolescence have a synergistic effect on lung function in early adulthood, consistent with the hypothesis that impaired prenatal pulmonary development is a risk factor for an accelerated decline of lung function leading to COPD in adulthood. In our mouse model, the combined effect did not appear to be mediated by an effect of prenatal or adolescent CS exposure on early pulmonary senescence. Our findings support the need for additional research on the molecular mechanisms involved in airway epithelial cell differentiation and alveolar formation in the early stages of life.

## Supplemental Material

(1.1 MB) PDF

## References

[r1] AIHW (Australian Institute of Health and Welfare) (2013). Tobacco smoking in the general population. In: *National Drug Strategy Household Survey: Detailed Report 2013*.. http://www.aihw.gov.au/WorkArea/DownloadAsset.aspx?id=60129549848.

[r2] Albrecht E Sillanpää E Karrasch S Alves AC Codd V Hovatta I 2014 Telomere length in circulating leukocytes is associated with lung function and disease. Eur Respir J 43 983 992, doi:10.1183/09031936.00046213 24311771

[r3] Bates SH Stearns WH Dundon TA Schubert M Tso AWK Wang Y 2003 STAT3 signalling is required for leptin regulation of energy balance but not reproduction. Nature 421 856 859, doi:10.1038/nature01388 12594516

[r4] Bentur L Hellou E Goldbart A Pillar G Monovich E Salameh M 2014 Laboratory and clinical acute effects of active and passive indoor group water-pipe (narghile) smoking. Chest 145 803 809, doi:10.1378/chest.13-0960 24158379

[r5] Biselli PJC Lopes FDTQS Moriya HT Rivero DHRF Toledo AC Saldiva PHN 2011 Short-term exposure of mice to cigarette smoke and/or residual oil fly ash produces proximal airspace enlargements and airway epithelium remodeling. Braz J Med Biol Res 44 460 468, doi:10.1590/S0100-879X2011007500040 21445523

[r6] Blacquière MJ Timens W Melgert BN Geerlings M Postma DS Hylkema MN 2009 Maternal smoking during pregnancy induces airway remodelling in mice offspring. Eur Respir J 33 1133 1140, doi:10.1183/09031936.00129608 19129273

[r7] Boucherat O Morissette MC Provencher S Bonnet S Maltais F 2016 Bridging lung development with chronic obstructive pulmonary disease. Relevance of developmental pathways in chronic obstructive pulmonary disease pathogenesis. Am J Respir Crit Care Med 193 362 375, doi:10.1164/rccm.201508-1518PP 26681127

[r8] Bureau MA, Monette J, Shapcott D, Paré C, Mathieu JL, Lippé J (1982). Carboxyhemoglobin concentration in fetal cord blood and in blood of mothers who smoked during labor.. Pediatrics.

[r9] Butler NR, Goldstein H, Ross EM (1972). Cigarette smoking in pregnancy: its influence on birth weight and perinatal mortality.. Br Med J.

[r10] Chen ZH Lam HC Jin Y Kim HP Cao J Lee SJ 2010 Autophagy protein microtubule-associated protein 1 light chain-3B (LC3B) activates extrinsic apoptosis during cigarette smoke-induced emphysema. Proc Natl Acad Sci U S A 107 18880 18885, doi:10.1073/pnas.1005574107 20956295 PMC2973911

[r11] Chipuk JE Kuwana T Bouchier-Hayes L Droin NM Newmeyer DD Schuler M 2004 Direct activation of Bax by p53 mediates mitochondrial membrane permeabilization and apoptosis. Science 303 1010 1014, doi:10.1126/science.1092734 14963330

[r12] Destro A Bianchi P Alloisio M Laghi L Di Gioia S Malesci A 2004 *K-ras* and *p16^INK4A^* alterations in sputum of NSCLC patients and in heavy asymptomatic chronic smokers. Lung Cancer 44 23 32, doi:10.1016/j.lungcan.2003.10.002 15013580

[r13] Drummond MB Hansel NN Connett JE Scanlon PD Tashkin DP Wise RA 2012 Spirometric predictors of lung function decline and mortality in early chronic obstructive pulmonary disease. Am J Respir Crit Care Med 185 1301 1306, doi:10.1164/rccm.201202-0223OC 22561963 PMC5448583

[r14] Eisner MD Anthonisen N Coultas D Kuenzli N Perez-Padilla R Postma D 2010 An official American Thoracic Society public policy statement: novel risk factors and the global burden of chronic obstructive pulmonary disease. Am J Respir Crit Care Med 182 693 718, doi:10.1164/rccm.200811-1757ST 20802169

[r15] Elliot JG Carroll NG James AL Robinson PJ 2003 Airway alveolar attachment points and exposure to cigarette smoke *in utero*. Am J Respir Crit Care Med 167 45 49, doi:10.1164/rccm.2110005 12502475

[r16] Fallica J Boyer L Kim B Serebreni L Varela L Hamdan O 2014 Macrophage migration inhibitory factor is a novel determinant of cigarette smoke–induced lung damage. Am J Respir Cell Mol Biol 51 94 103, doi:10.1165/rcmb.2013-0371OC 24490973 PMC4091857

[r17] Fuller E, ed (2013). *Smoking, Drinking and Drug Use among Young People in England in 2011*.. http://content.digital.nhs.uk/catalogue/PUB06921/smok-drin-drug-youn-peop-eng-2011-rep2.pdf.

[r18] Guerra S Stern DA Zhou M Sherrill DL Wright AL Morgan WJ 2013 Combined effects of parental and active smoking on early lung function deficits: a prospective study from birth to age 26 years. Thorax 68 1021 1028, doi:10.1136/thoraxjnl-2013-203538 23847259 PMC4706750

[r19] Hayatbakhsh MR Sadasivam S Mamun AA Najman JM Williams GM O’Callaghan MJ 2009 Maternal smoking during and after pregnancy and lung function in early adulthood: a prospective study. Thorax 64 810 814, doi:10.1136/thx.2009.116301 19525264

[r20] Hollams EM de Klerk NH Holt PG Sly PD 2014 Persistent effects of maternal smoking during pregnancy on lung function and asthma in adolescents. Am J Respir Crit Care Med 189 401 407, doi:10.1164/rccm.201302-0323OC 24251622

[r21] Ito K Barnes PJ 2009 COPD as a disease of accelerated lung aging. Chest 135 173 180, doi:10.1378/chest.08-1419 19136405

[r22] Janssens JP, Pache JC, Nicod LP (1999). Physiological changes in respiratory function associated with ageing.. Eur Respir J.

[r23] Kile ML Baccarelli A Tarantini L Hoffman E Wright RO Christiani DC 2010 Correlation of global and gene-specific DNA methylation in maternal-infant pairs. PLoS One 5 e13730, doi:10.1371/journal.pone.0013730 21060777 PMC2966409

[r24] Klar J Blomstrand P Brunmark C Badhai J Håkansson HF Brange CS 2011 Fibroblast growth factor 10 haploinsufficiency causes chronic obstructive pulmonary disease. J Med Genet 48 705 709, doi:10.1136/jmedgenet-2011-100166 21742743

[r25] Knudsen L Weibel ER Gundersen HJG Weinstein FV Ochs M 2010 Assessment of air space size characteristics by intercept (chord) measurement: an accurate and efficient stereological approach. J Appl Physiol (1985) 108 412 421, doi:10.1152/japplphysiol.01100.2009 19959763

[r26] Kotecha SJ Edwards MO Watkins WJ Henderson AJ Paranjothy S Dunstan FD 2013 Effect of preterm birth on later FEV1: a systematic review and meta-analysis. Thorax 68 760 766, doi:10.1136/thoraxjnl-2012-203079 23604458

[r27] Krishnamurthy J Torrice C Ramsey MR Kovalev GI Al-Regaiey K Su L 2004 *Ink4a*/*Arf* expression is a biomarker of aging. J Clin Invest 114 1299 1307, doi:10.1172/JCI22475 15520862 PMC524230

[r28] Lange P Celli B Agustí A Boje Jensen G Divo M Faner R 2015 Lung-function trajectories leading to chronic obstructive pulmonary disease. N Engl J Med 373 111 122, doi:10.1056/NEJMoa1411532 26154786

[r29] Livak KJ Schmittgen TD 2001 Analysis of relative gene expression data using real-time quantitative PCR and the 2(–Delta Delta C(T)) method. Methods 25 402 408, doi:10.1006/meth.2001.1262 11846609

[r30] Lødrup Carlsen KC, Jaakkola JJ, Nafstad P, Carlsen KH (1997). In utero exposure to cigarette smoking influences lung function at birth.. Eur Respir J.

[r31] Lopez AD Shibuya K Rao C Mathers CD Hansell AL Held LS 2006 Chronic obstructive pulmonary disease: current burden and future projections. Eur Respir J 27 397 412, doi:10.1183/09031936.06.00025805 16452599

[r32] Malkusch W Rehn B Bruch J 1995 Advantages of Sirius Red staining for quantitative morphometric collagen measurements in lungs. Exp Lung Res 21 67 77, doi:10.3109/01902149509031745 7537210

[r33] Maritz GS Harding R 2011 Life-long programming implications of exposure to tobacco smoking and nicotine before and soon after birth: evidence for altered lung development. Int J Environ Res Public Health 8 875 898, doi:10.3390/ijerph8030875 21556184 PMC3083675

[r34] Ng SP Conklin DJ Bhatnagar A Bolanowski DD Lyon J Zelikoff JT 2009 Prenatal exposure to cigarette smoke induces diet- and sex-dependent dyslipidemia and weight gain in adult murine offspring. Environ Health Perspect 117 1042 1048, doi:10.1289/ehp.0800193 19654910 PMC2717127

[r35] Ng SP Silverstone AE Lai ZW Zelikoff JT 2006 Effects of prenatal exposure to cigarette smoke on offspring tumor susceptibility and associated immune mechanisms. Toxicol Sci 89 135 144, doi:10.1093/toxsci/kfj006 16207940

[r36] Niewoehner DE, Kleinerman J (1974). Morphologic basis of pulmonary resistance in the human lung and effects of aging.. J Appl Physiol.

[r37] Parameswaran H Majumdar A Ito S Alencar AM Suki B 2006 Quantitative characterization of airspace enlargement in emphysema. J Appl Physiol (1985) 100 186 193, doi:10.1152/japplphysiol.00424.2005 16166240

[r38] Penn AL Rouse RL Horohov DW Kearney MT Paulsen DB Lomax L 2007 *In utero* exposure to environmental tobacco smoke potentiates adult responses to allergen in BALB/c mice. Environ Health Perspect 115 548 555, doi:10.1289/ehp.9780 17450223 PMC1852677

[r39] Pojer R, Whitfield JB, Poulos V, Eckhard IF, Richmond R, Hensley WJ (1984). Carboxyhemoglobin, cotinine, and thiocyanate assay compared for distinguishing smokers from non-smokers.. Clin Chem.

[r40] Rennard SI Drummond MB 2015 Early chronic obstructive pulmonary disease: definition, assessment, and prevention. Lancet 385 1778 1788, doi:10.1016/S0140-6736(15)60647-X 25943942 PMC4819246

[r41] Rinaldi M Maes K De Vleeschauwer S Thomas D Verbeken EK Decramer M 2012 Long-term nose-only cigarette smoke exposure induces emphysema and mild skeletal muscle dysfunction in mice. Dis Model Mech 5 333 341, doi:10.1242/dmm.008508 22279084 PMC3339827

[r42] Russell MA, Feyerabend C, Cole PV (1976). Plasma nicotine levels after cigarette smoking and chewing nicotine gum.. Br Med J.

[r43] Scherle W (1970). A simple method for volumetry of organs in quantitative stereology.. Mikroskopie.

[r44] Singh SP Gundavarapu S Smith KR Chand HS Saeed AI Mishra NC 2013 Gestational exposure of mice to secondhand cigarette smoke causes bronchopulmonary dysplasia blocked by the nicotinic receptor antagonist mecamylamine. Environ Health Perspect 121 957 964, doi:10.1289/ehp.1306611 23757602 PMC3734504

[r45] Stern DA Morgan WJ Wright AL Guerra S Martinez FD 2007 Poor airway function in early infancy and lung function by age 22 years: a non-selective longitudinal cohort study. Lancet 370 758 764, doi:10.1016/S0140-6736(07)61379-8 17765525 PMC2831283

[r46] Stick SM, Burton PR, Gurrin L, Sly PD, LeSouëf PN (1996). Effects of maternal smoking during pregnancy and a family history of asthma on respiratory function in newborn infants.. Lancet.

[r47] Suter MA Anders AM Aagaard KM 2013 Maternal smoking as a model for environmental epigenetic changes affecting birthweight and fetal programming. Mol Hum Reprod 19 1 6, doi:10.1093/molehr/gas050 23139402 PMC3521486

[r48] Svanes C, Omenaas E, Jarvis D, Chinn S, Gulsvik A, Burney P (2004). Parental smoking in childhood and adult obstructive lung disease: results from the European Community Respiratory Health Survey.. Thorax.

[r49] Tomioka S Bates JHT Irvin CG 2002 Airway and tissue mechanics in a murine model of asthma: alveolar capsule vs. forced oscillations. J Appl Physiol (1985) 93 263 270, doi:10.1152/japplphysiol.01129.2001 12070213

[r50] Tsuji T Aoshiba K Nagai A 2004 Cigarette smoke induces senescence in alveolar epithelial cells. Am J Respir Cell Mol Biol 31 643 649, doi:10.1165/rcmb.2003-0290OC 15333326

[r51] Tsuji T Aoshiba K Nagai A 2006 Alveolar cell senescence in patients with pulmonary emphysema. Am J Respir Crit Care Med 174 886 893, doi:10.1164/rccm.200509-1374OC 16888288

[r52] Tyner SD Venkatachalam S Choi J Jones S Ghebranious N Igelmann H 2002 p53 mutant mice that display early ageing-associated phenotypes. Nature 415 45 53, doi:10.1038/415045a 11780111

[r53] Upton MN Smith GD McConnachie A Hart CL Watt GCM 2004 Maternal and personal cigarette smoking synergize to increase airflow limitation in adults. Am J Respir Crit Care Med 169 479 487, doi:10.1164/rccm.200211-1357OC 14630616

[r54] van Deursen JM 2014 The role of senescent cells in ageing. Nature 509 439 446, doi:10.1038/nature13193 24848057 PMC4214092

[r55] Van Durme YMTA Eijgelsheim M Joos GF Hofman A Uitterlinden AG Brusselle GG 2010 Hedgehog-interacting protein is a COPD susceptibility gene: the Rotterdam Study. Eur Respir J 36 89 95, doi:10.1183/09031936.00129509 19996190

[r56] Vanoirbeek JAJ Rinaldi M De Vooght V Haenen S Bobic S Gayan-Ramirez G 2010 Noninvasive and invasive pulmonary function in mouse models of obstructive and restrictive respiratory diseases. Am J Respir Cell Mol Biol 42 96 104, doi:10.1165/rcmb.2008-0487OC 19346316

[r57] Wongtrakool C Wang N Hyde DM Roman J Spindel ER 2012 Prenatal nicotine exposure alters lung function and airway geometry through α7 nicotinic receptors. Am J Respir Cell Mol Biol 46 695 702, doi:10.1165/rcmb.2011-0028OC 22246862 PMC3359906

[r58] Wu ZX Hunter DD Kish VL Benders KM Batchelor TP Dey RD 2009 Prenatal and early, but not late, postnatal exposure of mice to sidestream tobacco smoke increases airway hyperresponsiveness later in life. Environ Health Perspect 117 1434 1440, doi:10.1289/ehp.0800511 19750110 PMC2737022

[r59] Zhou S Wright JL Liu J Sin DD Churg A 2013 Aging does not enhance experimental cigarette smoke-induced COPD in the mouse. PloS One 8 e71410, doi:10.1371/journal.pone.0071410 23936505 PMC3732225

