# Supplementary material for: Combined Effects of in Utero and Adolescent Tobacco Smoke Exposure on Lung Function in C57Bl/6J Mice
Source: Environ Health Perspect. 2016 Nov 4;125(3):392–9. doi: 10.1289/EHP54 (PMC5332197; doi:10.1289/EHP54)
Supplement: (1.1 MB) PDF [file EHP54.s001.acco.pdf]

Environ Health Perspect
DOI: 10.1289/EHP54

Note to readers with disabilities: *EHP* strives to ensure that all journal content is accessible to all readers. However, some figures and Supplemental Material published in *EHP* articles may not conform to 508 standards due to the complexity of the information being presented. If you need assistance accessing journal content, please contact ehponline@niehs.nih.gov. Our staff will work with you to assess and meet your accessibility needs within 3 working days.

Supplemental Material

Combined Effects of *in Utero* and Adolescent Tobacco Smoke Exposure on Lung Function in C57B1/6J Mice

David Drummond, Mélissa Baravalle-Einaudi, Guillaume Lezmi, Shamila Vibhushan, Marie-Laure Franco-Montoya, Alice Hadchouel, Jorge Boczkowski, and Christophe Delacourt

Table of Contents

Lung Immunohistochemistry
Algorithms used with Image J software
Table S1: HbCO levels in dams and pups following cigarette smoke or room air exposure.
Table S2: Primer sequences used in the study
Table S3: Maternal weights from pre-conception to delivery.
Table S4: Pup weights from day 0 to day 49.
Table S5: Lung mechanics on days 21 and 49
Table S6: Lung mechanics on days 21 and 49 (values normalised against the air and air-air groups)
Table S7: Lung mechanics on D21, by sex
Table S8: Lung mechanics on D49, by sex.
Figure S1: Flow chart of mice during experiment 1
Figure S2: Flow chart of mice during experiment 2
Figure S3: Immunostaining of p16 in alveoli at day 21.

Lung immunohistochemistry

Paraffin-embedded lung sections were deparaffinized with xylene, rehydrated gradually with graded alcohol solutions, and then washed with deionized water. Antigens were unmasked by boiling for 20 minutes in 10 mM citric acid buffer pH 6, (Sigma). After cooling at room temperature (RT), sections were rinsed in phosphate-buffered saline (PBS), pH 7.4 (Life technologies), and blocked with 2.5% normal horse serum (RTU Vectastain) for 15 minutes at RT. Then, sections were incubated overnight at 4°C in an humidified chamber with a diluted anti-p16 rabbit polyclonal antibody (1:1000, PA5-20379, ThermoFisher). After overnight incubation, sections were washed in PBS and endogenous peroxidase was quenched with 3% H202 for 20 minutes at RT. Sections were then incubated with biotinylated universal secondary antibody (RTU Vectastain, Vector Laboratories) for 10 minutes at RT, followed by and incubation with the streptavidin-peroxidase complex (RTU Vectastain) for 5 minutes at RT. After further washing, chromogen 3,3′-diaminobenzidine tetrachloride/nickel-cobalt substrate was applied, yielding a brown colored reaction product, and sections were counterstained with hematoxylin (Hematoxylin QS, Vector Laboratories, H-3404).

Algorithms used with Image J software
(National Institutes of Health, http://rsb.info.nih.gov/nih-image)

A. Red Sirius in bronchus

```
run("Colour Deconvolution", "vectors=[User values] [r1]=0.119          [g1]=0.613 [b1]=0.781 [r2]=0.55 [g2]=0.46
[b2]=0.69 [r3]=0.41 [g3]=0.45 [b3]=0.797");;
//run("Threshold...");
setThreshold(0, 110);
run("Measure");
close();
setAutoThreshold("Default");
setThreshold(0, 140);
run("Measure");
run("Close All");
```

B . p16 expression in lung parenchyma

```
run("Colour Deconvolution", "vectors=[User values] [r1]=0.486 [g1]=0.547 [b1]=0.682 [r2]=0.508 [g2]=0.670
[b2]=0.541 [r3]=0.377 [g3]=0.622 [b3]=0.686");
setAutoThreshold("Default");
//run("Threshold...");
setThreshold(0, 150);
run("Measure");
close();
setAutoThreshold("Default");
setThreshold(0, 150);
run("Measure");
close();
run("Close All");
open("");
```

Table S1: HbCO levels in dams and pups following cigarette smoke or room air exposure.

Experiment	Dams Pre-conception day 10		Dams Gestational day 5		Pups Day 23		Pups Day 48	
	Smoke n=5	Air n=5	Smoke n=5	Air n=5	Smoke n=5	Air n=5	Smoke n=5	Air n=5
Experiment 1	32.5 (25.0-38.3)	1.3 (1.2-2.2)	20.9 (9.1-35.9)	0.7 (0.5-1.7)				
Experiment 2	21.4 (15.2-26.1)	0.8 (0.5-1.2)			24.1 (12.1-29.5)	0.7 (0.5-1.1)	31.2 (24.7-41.7)	1.1 (0.9-1.9)

Results are presented as median with their interquartile range in brackets. Exposure to CS was monitored through recurrent measurements of blood HbCO levels in dams before mating (pre-conception day 10), during pregnancy (gestational day 5), and in pups two days after the beginning (day 23) and the day before the end (day 48) of the exposure period. The HbCO levels at gestational day 5 during experiment 2 were not measured due to technical problems.

Table S2: Primer sequences used in the study

Gene	Oligonucleotide sequence
**Sf3a1**	F: CCACTGAGTCCAAACAGCCAAT
	R: AGCTTCAAATTCAGGCCCAT
**Bax**	F: CGGCGAATTGGAGATGAACT
	R: GCAAAGTAGAAGAGGGCAACCA
**p53**	F: GGCCCCTGTCATCTTTTGTC
	R: TGGAGGTGTGGCGCTGA
**p21**	F: GAACATCTCAGGGCCGAAAA
	R: CGTGGGCACTTCAGGGTTT
**P16**	F: CCCAACGCCCCGAACT
	R: GCAGAAGAGCTGCTACGTGAA
**HPRT1**	F: GTTAAGCAGTACAGCCCCAAAATG
	R: TCAAGGGCATATCCAACAACAAAC

Table S3: Maternal weights (g) from pre-conception to delivery, in mice exposed to tobacco smoke or to room air. No significant difference was observed between groups.

Experiment	Pre-conception Day 14		Gestational Day 0		Gestational Day 12		Gestational Day 18	
	Smoke	Air	Smoke	Air	Smoke	Air	Smoke	Air
Experiment 1								
Number	6	7	6	7	6	7	6	7
Median (IQR)	24.6 (23.4-25.4)	24.3 (22.4-25.7)	21.8 (20.5-23.3)	23.8 (23.0-25.4)	25.7 (24.2-26.5)	26.0 (25.2-27.8)	33.8 (27.4-34.6)	34.1 (32.44-35.11)
Experiment 2								
Number	7	7	7	7	7	7	7	7
Median (IQR)	24.6 (24.4-25.5)	24.9 (23.3-25.5)	23.7 (23.1-24.2)	24.3 (23.7-24.9)	26.3 (24.6-26.8)	26.8 (25.6-28.5)	32.2 (31.6-33.3)	32.9 (32.4-35.3)

DOI: 10.1289/EHP54

Table S4: Pup weights (g) from day 0 to day 49

Exposure Group	D0 (Birth)		D21		D49	
	N	Median (IQR)	N	Median (IQR)	N	Median (IQR)
Experiment 1						
Smoke	31	1.1 (1.1–1.2)	11	9.4 (7.5–9.8)	NA	NA
Air	29	1.3* (1.3–1.4)	11	9.3 (7.8–9.7)	NA	NA
Experiment 2, pups euthanized D21						
Smoke	NA	NA	14	11.3 (10.0-13.0)	NA	NA
Air	NA	NA	15	11.7 (11.1-12.5)	NA	NA
Experiment 2, pups euthanized D49						
SS	NA	NA	11	9.7 (7.5-10.7)	11	16.2**, *** (15.5-17.1)
SA	NA	NA	11	10.0 (6.4-11.1)	11	17.2 (16.5-20.8)
AS	NA	NA	19	10.9 (9.8-11.7)	19	18.5 (16.9-20.0)
AA	NA	NA	12	11.2 (8.4-12.6)	12	18.2 (16.6-21.4)
Total						
Smoke	NA	NA	47	10.0 (8.6-11.3)	NA	NA
Air	NA	NA	57	10.7 (9.4-11.7)	NA	NA

p=0.02 for overall comparison (Kruskall-Wallis test) on Day 49. *: p<0.001 for the Smoke versus Air comparison. **: p<0.05 for the SS versus AA comparison. ***: p<0.05 for the SS versus AS comparison.
NA: Not available

Environ Health Perspect
DOI: 10.1289/EHP54

Table S5: Lung mechanics on days 21 and 49

Perturbation	D21 *In utero* exposure to			D49 *In utero* + post-natal exposure to air (A) and/or smoke (S)				
	Air (A) *n*=15	Smoke (S) *n*=14	*p*	AA *n*=8	AS *n*=8	SA *n*=8	SS *n*=8	*p*
Weight (g)	11.7 (11.1-12.5)	11.3 (10.0-13.0)	0.49	20.2 (16.6-22.8)	17.9 (16.0-19.6)	19.0 (16.9-21.3)	16.2 (15.6-18.1)	**0.04**
Single compartment (Snapshot)								
Resistance ($cmH_2O.s/ml$)	1.31 (1.16-1.76)	1.51 (1.35-1.65)	0.47	0.8 (0.6-1.2)	0.7 (0.7-0.8)	0.8 (0.8-1.0)	0.9 (0.8-1.1)	0.24
Elastance (cmH_2O/ml)	53.9 (49.3-58.5)	62.5 (57.7-69.9)	**<0.01**	38.3 (33.4-39.2)	35.4 (33.8-45.6)	41.3 (40.0-50.8)	51.8[a.b] (44.1-60.3)	**<0.01**
Compliance (ml/cmH_2O)	0.019 (0.017-0.020)	0.016 (0.015-0.017)	**0.01**	0.030 (0.026-0.032)	0.030 (0.028-0.032)	0.025 (0.024-0.029)	0.022[a.b] (0.019-0.025)	**<0.01**
Constant-phase model (forced oscillation)								
Airway resistance (Rn) ($cmH_2O.s/ml$)	0.67 (0.56-0.86)	0.74 (0.59-0.85)	0.4	0.34 (0.30-0.47)	0.35 (0.31-0.39)	0.42 (0.38-0.46)	0.42 (0.37-0.51)	0.11
Tissue damping (G) (cmH_2O/ml)	10.2 (8.4-11.1)	10.4 (9.0-11.9)	0.35	5.9 (5.3-7.3)	6.0 (5.7-7.1)	6.4 (6.3-7.1)	8.0 (6.6-8.6)	0.06
Tissue elasticity (H) (cmH_2O/ml)	52.6 (50.4-64.8)	61.8 (57.9-69.8)	**0.02**	36,3 (32,7-39,9)	33.3 (31.6-34.7)	42.0 (39.6-44.3)	46.0[a.b] (43.9-53.7)	**<0.01**
Pressure-volume loops (ramp volume)								
Static compliance (ml/cmH_2O)	0.047 (0.044-0.049)	0.041 (0.038-0.045)	**<0.01**	0.070 (0.067-0.082)	0.078 (0.068-0.084)	0.070 (0.061-0.079)	0.060[b] (0.050-0.065)	**<0.05**
Static elastance (cmH_2O/ml)	21.3 (20.4-22.6)	24.3 (22.3-26.2)	**<0.01**	14.3 (12.2-14.9)	12.8 (11.9-14.6)	14.2 (12.8-16.3)	16.7[b] (15.5-19.9)	**<0.05**

Median values and interquartile ranges. a: $p<0.05$ for the SS versus AA comparison. b: $p<0.05$ for the SS versus AS comparison.
AA: mice never exposed to cigarette smoke. AS: mice exposed only during postnatal life. SA: mice exposed only during prenatal development. SS: mice exposed during both prenatal development and postnatal life.

Table S6: Lung mechanics on days 21 and 49 (values normalised against the air and air-air groups)

Perturbation	D21 *In utero* exposure to			D49 *In utero* + post-natal exposure to air (A) and/or smoke (S)				
	Air (A) *n*=15	Smoke (S) *n*=14	*p*	AA *n*=8	AS *n*=8	SA *n*=8	SS *n*=8	*p*
Single compartment (Snapshot)								
Resistance	100 (88-135)	115 (103-126)	0.47	100 (76-148)	89 (82-102)	101 (94-124)	110 (99-134)	0.24
Elastance	100 (92-109)	116 (107-130)	**<0.01**	100 (92-115)	101 (94-106)	120 (103-124)	132[a.b] (121-155)	**<0.01**
Compliance	100 (84-110)	89 (79-101)	**0.01**	100 (87-108)	99 (94-107)	84 (81-97)	76[a.b] (65-83)	**<0.01**
Constant-phase model (forced oscillation)								
Airway resistance (Rn)	100 (78-120)	106 (91-114)	0.4	100 (89-138)	102 (93-115)	124 (112-135)	123 (109-150)	0.11
Tissue damping (G)	100 (83-109)	103 (89-117)	0.35	100 (91-123)	102 (96-121)	108 (107-121)	136 (112-146)	0.06
Tissue elasticity (H)	100 (96-123)	117 (110-133)	**0.02**	100 (90-110)	92 (87-96)	115 (109-122)	126[a.b] (121-148)	**<0.01**
Pressure-volume loops (ramp volume)								
Static compliance	100 (94-104)	88 (81-95)	**<0.01**	100 (96-117)	111 (98-120)	100 (88-112)	85[b] (72-92)	**<0.05**
Static elastance	100 (96-106)	114 (105-123)	**<0.01**	100 (85-104)	90 (83-101)	99 (89-114)	117[b] (109-140)	**<0.05**

Median values and interquartile ranges. a: $p<0.05$ for the SS versus AA comparison. b: $p<0.05$ for the SS versus AS comparison.
AA: mice never exposed to cigarette smoke. AS: mice exposed only during postnatal life. SA: mice exposed only during prenatal development. SS: mice exposed during both prenatal development and postnatal life.

Environ Health Perspect
DOI: 10.1289/EHP54

Table S7: Lung mechanics on D21, by sex

Perturbation	D21<br>*In utero* exposure to air (A) and/or smoke (S)			
	Air (A)		Smoke (S)	
	M<br>*n*=8	F<br>*n*=7	M<br>*n*=7	F<br>*n*=7
Single compartment (Snapshot)				
Resistance ($cmH_2O.s/ml$)	1.27<br>(1.12-1.9)	1.38<br>(1.16-1.76)	1.50<br>(1.41-1.63)	1.51<br>(1.28-1.71)
Elastance (cmH_2O/ml)	53.9<br>(49.7-65.4)	52.8<br>(48.7-58.2)	63.8<br>(60.4-68.3)	60.2<br>(56.5-73.9)
Compliance (ml/cmH_2O)	0.019<br>(0.015-0.020)	0.019<br>(0.017-0.020)	0.016<br>(0.015-0.017)	0.017<br>(0.014-0.018)
Constant-phase model (forced oscillation)				
Airway resistance (Rn) ($cmH_2O.s/ml$)	0.62<br>(0.53-0.86)	0.69<br>(0.66-0.86)	0.71<br>(0.60-0.85)	0.77<br>(0.56-0.82)
Tissue damping (G) (cmH_2O/ml)	9.6<br>(8.5-10.9)	10.2<br>(8.3-14.9)	10.0<br>(9.2-12.4)	10.9<br>(8.8-11.8)
Tissue elasticity (H) (cmH_2O/ml)	52.0<br>(50.5-63.2)	54.1<br>(43.9-73.0)	62.5<br>(60.6-72.4)	58.0<br>(57.8-68.4)
Pressure-volume loops (ramp volume)				
Static compliance (ml/cmH_2O)	0.047<br>(0.045-0.053)	0.047<br>(0.039-0.049)	0.042<br>(0.038-0.045)	0.040<br>(0.038-0.045)
Static elastance (cmH_2O/ml)	21.4<br>(18.8-22.4)	21.4<br>(20.4-25.9)	23.7<br>(22.4-26.5)	25.3<br>(22-26.1)

The results were not significantly different between male and female mice exposed to air, or between male and female mice exposed to smoke

Environ Health Perspect
DOI: 10.1289/EHP54

Table S8: Lung mechanics on D49, by sex.

Perturbation	D49<br>*In utero* + post-natal exposure to air (A) and/or smoke (S)							
	AA		AS		SA		SS	
	M<br>*n*=4	F<br>*n*=4	M<br>*n*=4	F<br>*n*=4	M<br>*n*=4	F<br>*n*=4	M<br>*n*=4	F<br>*n*=4
Single compartment (Snapshot)								
Resistance (cmH_2O.s/ml)	0.63<br>(0.61-1.29)	0.89<br>(0.66-1.21)	0.68<br>(0.66-0.74)	0.81<br>(0.69-0.96)	0.79<br>(0.71-1.03)	0.84<br>(0.81-1.02)	0.81<br>(0.72-0.98)	1.03<br>(0.87-1.19)
Elastance (cmH_2O/ml)	32.5<br>(29.9-36.4)	37.9<br>(33.4-39.1)	31.5<br>(29.4-33.3)	35.2<br>(34.1-42.9)	36<br>(27.8-40.6)	41.2<br>(39.0-48.4)	40.3<br>(40.1-44.2)	49.8<br>(43.5-58.7)
Compliance (ml/cmH_2O)	0.031<br>(0.027-0.033)	0.026<br>(0.026-0.030)	0.32<br>(0.30-0.034)	0.028<br>(0.024-0.029)	0.028<br>(0.025-0.036)	0.024<br>(0.021-0.026)	0.025<br>(0.023-0.025)	0.020<br>(0.017-0.023)
Constant-phase model (forced oscillation)								
Airway resistance (Rn) (cmH_2O.s/ml)	0.33<br>(0.32-0.47)	0.41<br>(0.27-0.64)	0.33<br>(0.29-0.35)	0.39<br>(0.33-0.44)	0.38<br>(0.33-0.46)	0.44<br>(0.41-0.53)	0.38<br>(0.33-0.41)	0.49<br>(0.43-0.55)
Tissue damping (G) (cmH_2O/ml)	5.8<br>(4.9-5.9)	7.2<br>(5.8-7.5)	6<br>(5.6-6.9)	6.4<br>(5.7-8.1)	6.3<br>(5.5-7.1)	6.6<br>(6.3-7.7)	7.5<br>(6.3-8.2)	8.4<br>(6.9-10.1)
Tissue elasticity (H) (cmH_2O/ml)	33.7<br>(29.9-36.3)	38.5<br>(33.8-40.7)	31.8<br>(28.1-33.6)	34.5<br>(32.9-45.4)	41.9<br>(35.2-44.3)	42.8<br>(40.2-51.6)	44.5<br>(41.7-45.8)	51.5<br>(46.3-62.7)
Pressure-volume loops (ramp volume)								
Static compliance (ml/cmH_2O)	0.081<br>(0.061-0.088)	0.069<br>(0.067-0.070)	0.083<br>(0.078-0.089)	0.069<br>(0.063-0.077)	0.076<br>(0.070-0.091)	0.062<br>(0.054-0.071)	0.062<br>(0.052-0.071)	0.057<br>(0.048-0.063)
Static elastance (cmH_2O/ml)	12.3<br>(11.4-16.8)	14.5<br>(14.3-14.9)	12.0<br>(11.3-12.9)	14.5<br>(13.0-15.9)	13.3<br>(11.0-14.3)	16.2<br>(14.2-18.8)	16.2<br>(14.2-19.4)	17.8<br>(15.8-20.7)

Statistical analysis was not performed because of the small number of animals in each group.

DOI: 10.1289/EHP54

Figure S1: Flow chart of mice during the first experiment

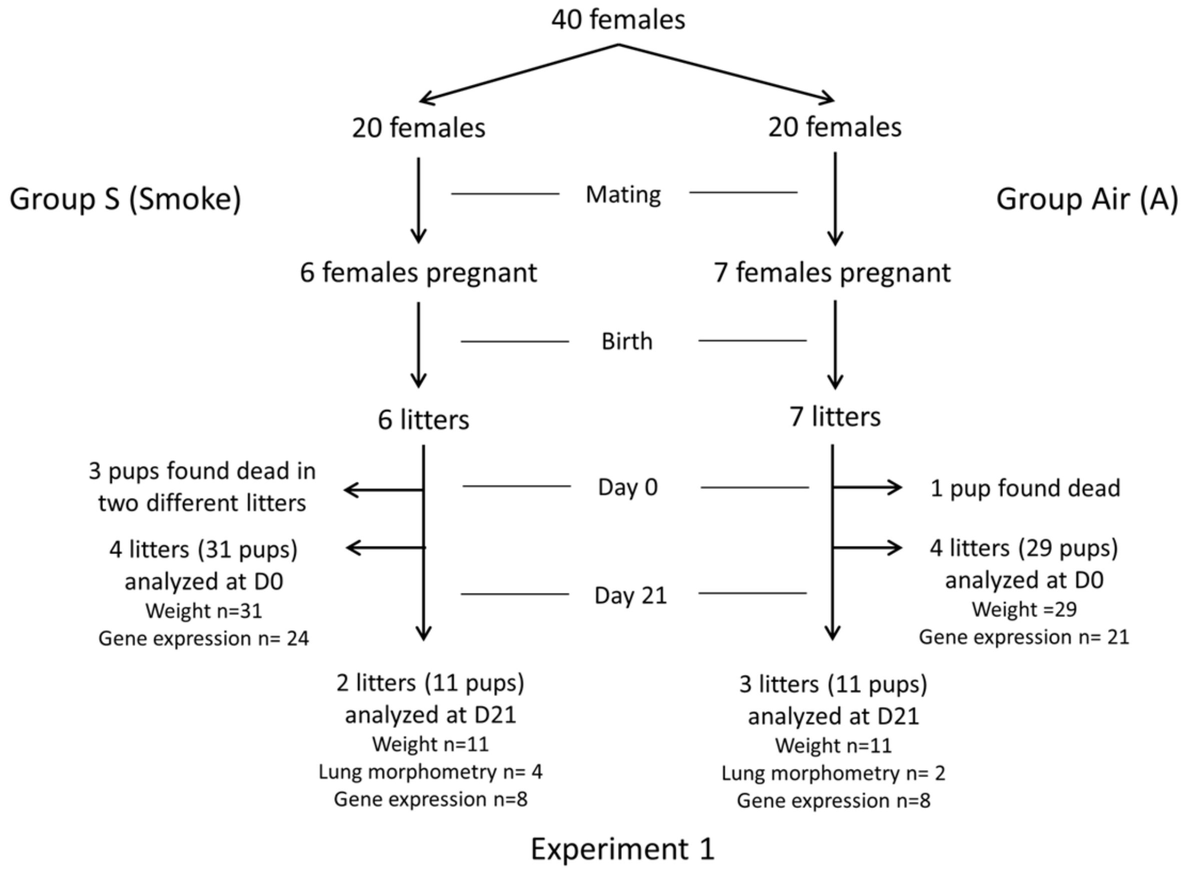

Figure S1: Flow chart of mice during the first experiment. D0: day 0. D21: day 21. At birth, three pups were found dead in the group S (smoke) and one in the group A (air).

Figure S2: Flow chart of mice during the second experiment

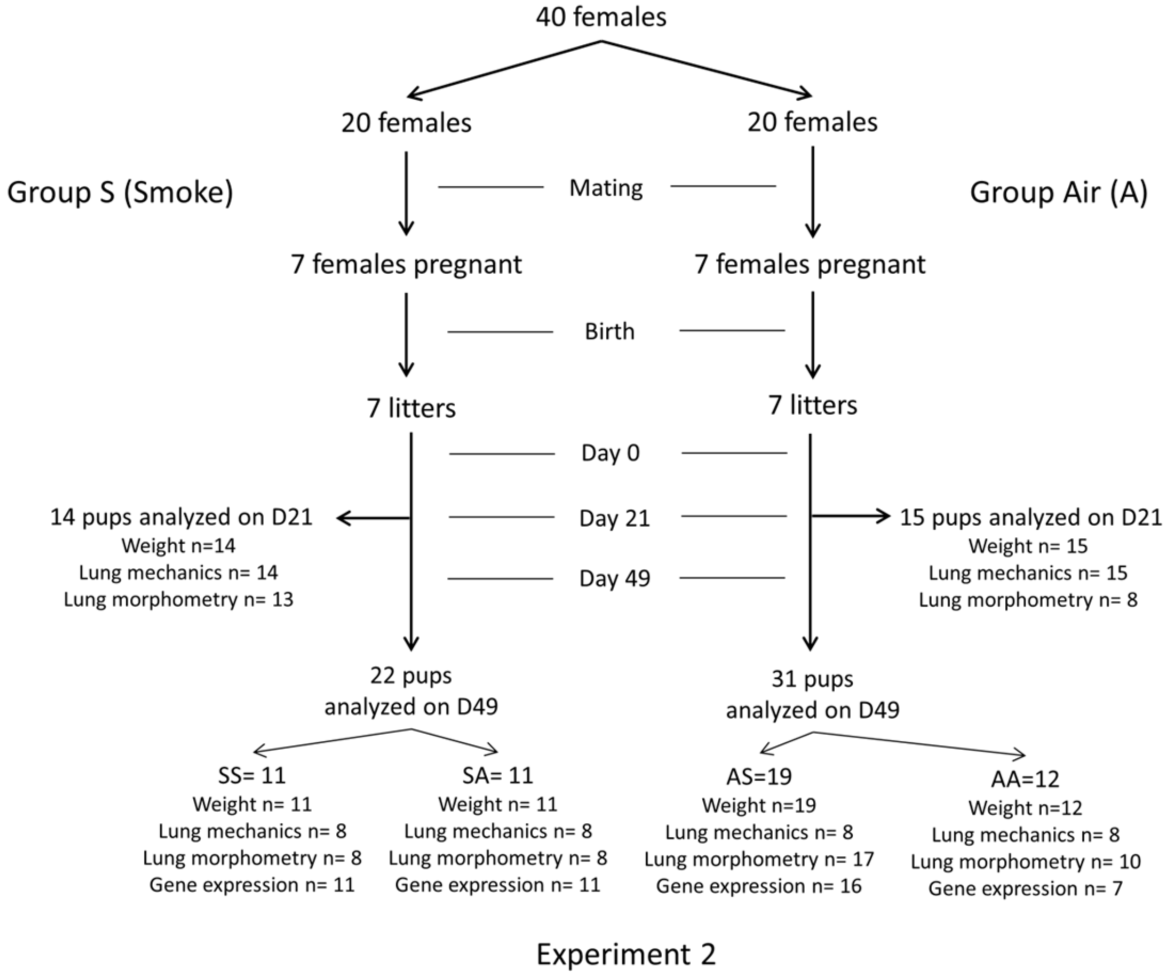

Figure S2: Flow chart of mice during the second experiment. D0: day 0. D21: day 21. D49: day 49. SS: Smoke-Smoke group. SA: Smoke-Air group. AS: Air-Smoke group. AA: Air-Air group.

Figure S3: p16 expression in lung parenchyma on day 21

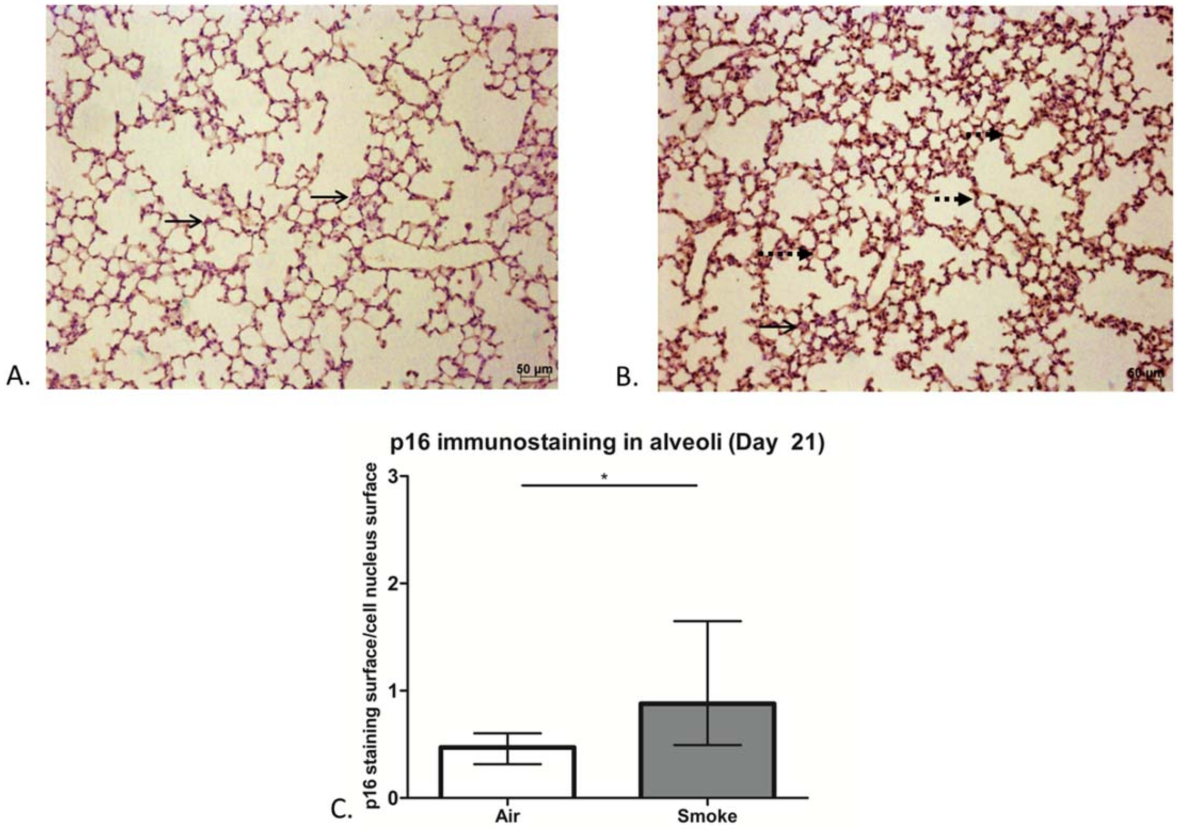

Figure S3: p16 protein expression in lung parenchyma of 21-day-old mice A. from the group Air. B. from the group Smoke.

p16 expression was visible in brown-dark (dashed arrows) and cell nucleus were visible in purple (plain arrows).

C. Quantification of p16 immunostaining surface normalized on cell nucleus surface. p16 expression was superior in mice exposed prenatally to CS compared to mice exposed to filtered room air (Smoke n=8, Air n=8, p=0.02). Results are presented as median and interquartile range.
